# A practical assessment of hamstring muscle endurance and fatigue using the maximum-speed single-leg bridge test

**DOI:** 10.1186/s13102-025-01458-y

**Published:** 2025-12-05

**Authors:** Yuto Sano, Masashi Kawabata, Bas Van Hooren, Yuki Sumiya, Masaki Murase, Yuto Watanabe, Yasuhisa Shimono, Tomonori Kenmoku, Hiroyuki Watanabe, Naonobu Takahira

**Affiliations:** 1 Department of Sports Medicine, Kitasato University Graduate School of Medical Sciences, Sagamihara, Japan; 2 Department of Rehabilitation, Yokohama Sports Medical Center, Yokohama, Japan; 3 Physical Therapy for Sports and Musculoskeletal System, Kitasato University Graduate School of Medical Sciences, Sagamihara, Japan; 4 Department of Rehabilitation, Kitasato University School of Allied Health Sciences, Sagamihara, Japan; 5 Department of Nutrition and Movement Sciences, NUTRIM Institute of Nutrition and Translational Research in Metabolism, Maastricht University Medical Centre+, Maastricht, The Netherlands; 6 Sagamihara Dynaboars, Mitsubishi Heavy Industries, Sagamihara, Japan; 7 Department of Orthopaedic Surgery, Kitasato University School of Medicine, Sagamihara, Japan

**Keywords:** Hamstring strain injury, Prevention, Rehabilitation, Surface electromyography

## Abstract

**Background:**

The single-leg bridge test aims to evaluate hamstring muscle endurance but lacks speed regulation and requires prolonged testing, which may compromise sensitivity and reliability. The maximum-speed single-leg bridge test (MS-SLBT) was developed to address these limitations, but its ability to induce hamstrings fatigue remains unclear. Furthermore, it is unknown which practical outcomes, such as buttock-raising height or speed, best reflect this fatigue. Therefore, we assessed changes in muscle activation, buttock-raising height, buttock-raising speed, and heel-bearing force during 20 repetitions of the MS-SLBT.

**Methods:**

This cross-sectional observational study included 26 male recreational athletes. Surface electromyography was used to assess fatigue by measuring the median frequency (MDF) and amplitude of the semitendinosus (ST), biceps femoris (BF), and gluteus maximus (GM). Motion characteristics, including heel-bearing force and buttock-raising height and speed, were analyzed across 18 repetitions (from the 2nd to the 19th) to evaluate changes during the MS-SLBT.

**Results:**

During the MS-SLBT, the MDF of the ST and BF decreased by 15.2% and 14.9%, respectively (*p* < 0.01), while the GM showed no significant change (− 2.4%, *p* = 0.57). The amplitude significantly increased after the 6th and 9th repetitions for ST and BF, respectively. Buttock-raising speed and heel-bearing force at the 19th repetition decreased by 8.9% and 10.4%, respectively, compared with the 2nd repetition (*p* < 0.01), while buttock-raising height did not significantly change (+ 1.1%, *p* = 0.73).

**Conclusions:**

Performing 20 MS-SLBT repetitions induced local hamstrings fatigue as indicated by MDF reductions and amplitude increases in the ST and BF. Buttock-raising speed and heel-bearing peak force decreased with fatigue and therefore can serve practical indicators of this fatigue, whereas buttock-raising height was not sensitive to fatigue.

## Introduction

Sports that involve frequent sprinting have a high incidence of hamstring strain injuries (HSI) [1–3]. Re-injury occurs in 18–33% of the individuals, with most injuries occurring within 2 months of return to play [3, 4]. Despite numerous studies, prevention strategies remain inadequate, as evidenced by the continued rise in incidence observed from the 2001/02 to 2021/22 seasons in elite football [3].

Reduced hamstring muscle endurance—the ability of the hamstring muscles to sustain submaximal force output—has been reported to be associated with a higher HSI re-injury risk [5]. However, in this previous study, hamstring muscle endurance was investigated using a repeated prone leg curl with the hip at 0° of flexion, and an angular velocity at the knee joint of approximately 135°/s (based on a movement tempo of 90 bpm and a range of motion of about 90°) [5]. This assessment differs from sprinting in the joint angles and joint angular velocities, and therefore likely evaluates the hamstrings at shorter fascicle lengths and lower contraction velocities. These differences may reduce the sensitivity of this test to assess muscle endurance in the context of sprinting and its risk of HSI. Indeed, HSI occur primarily during the late swing or early stance phases of high-speed sprinting [6–8], where hip and knee flexion angles range from 40° to 60° and 5° to 30°, respectively [9]. As sprinting speed increases, greater demands are placed on the hamstrings [10, 11], with the hip extension angular velocity exceeding 650°/s and the knee extension angular velocity surpassing 1,000°/s [12].

The single-leg bridge test (SLBT) represents an alternative test to evaluate hamstring muscle endurance [13]. It involves a repetitive buttock-raising movement performed until failure, starting from a position with approximately 45–60° of hip flexion and 20° of knee flexion, which closely resembles the joint angles observed in the late swing phase of sprinting. A lower number of repetitions performed during the SLBT has been prospectively associated with a higher risk of HSI [13]. Similarly, lower isometric heel-bearing force measured in a comparable position has been reported to be associated with HSI [14]. However, in the former study, the association between the number of repetitions performed during the SLBT and HSI was observed only for the right leg in univariate analysis, with multivariate analysis showing no significant predictive effect of the number of repetitions in the SLBT for HSI [13]. In the latter study, although lower isometric heel-bearing force was associated with HSI, the proposed cut-off value showed low specificity and limited predictive validity [14]. These inconsistent results may be because the SLBT lacks regulation of movement speed, which is essential for targeting fast-twitch fibers of the hamstrings. Specifically, although slow-speed tasks to exhaustion may eventually recruit these fast-twitch fibers, their activation tends to occur later and at lower levels, making high-speed, high-intensity movements more effective for engaging these fibers [15, 16]. Indeed, the hamstrings contain a high proportion of Type II fibers, with ST comprising 67% and BF 53% [17, 18]. This predominance of Type II fibers may contribute to reduced fatigue resistance [19]. Further, a recent MRI-based study focusing specifically on the hamstrings demonstrated that a higher proportion of Type II fibers was also associated with an increased risk of HSI [20].

The maximum-speed single-leg bridge test (MS-SLBT) may overcome some limitations of the SLBT. Specifically, it combines a relatively high hip extension velocity, as seen during the late swing phase of sprinting with the SLBT’s advantages—namely, endurance assessment, biarticular movement, and joint positions mimicking those seen during the occurrence of HSI [21]. Moreover, the MS-SLBT has been shown to elicit approximately 15% greater muscle activation in the semitendinosus (ST) and biceps femoris (BF) compared to the SLBT [21]. With absolute activation levels exceeding 90% of the maximum voluntary isometric contraction (MVIC), the MS-SLBT therefore better reflects the demands of sprinting [21]. Further, in contrast to the SLBT, which requires repetitions until failure, the MS-SLBT uses a standardized protocol consisting of 20 repetitions [21]. This defined endpoint of 20 repetitions has been chosen primarily to reduce variability. Indeed, in the SLBT, participants may continue despite deteriorating movement quality or despite a considerably slower speed, leading to inconsistent test termination. The MS-SLBT addresses this limitation by standardizing the endpoint to 20 repetitions, ensuring a more reliable assessment. The 20 repetitions cut-off is chosen based on findings that the injured side in the SLBT typically reaches exhaustion between 20 and 27 repetitions [13]. It is however unknown whether 20 repetitions are sufficient to elicit fatigue and thus for assessing hamstring muscle endurance capacity.

The median frequency (MDF) of surface electromyography (sEMG) muscle activation is a well-established indicator of local muscle fatigue [22], where local muscle fatigue can be defined as an inability of a specific muscle to sustain force production. In a field setting, sEMG is however typically not available because it requires specialized equipment and multiple preparation steps, including precise electrode placement and skin preparation [23], thereby necessitating the use of other indicators of fatigue. Although changes in buttock-raising height, buttock-raising speed, or heel-bearing force have been suggested to reflect fatigue-related performance decline during the MS-SLBT, their validity has not been firmly established. By combining muscle activation and movement-based parameters, this study aimed to 1) determine whether the standardized protocol of the MS-SLBT induces hamstring fatigue and to 2) identify appropriate practical movement-based indicators for muscle endurance assessment. We hypothesized that 20 MS-SLBT repetitions would induce measurable fatigue in the hamstring muscles, which would be reflected by changes in muscle activation and a reduction in movement speed.

## Methods

### Study design

In this cross-sectional observational study, we investigated hamstring fatigue during the MS-SLBT to identify potential movement-based indicators for muscle endurance assessment. Participants performed the MS-SLBT while muscle activation, buttock-raising height and speed, and heel-bearing force were measured. The recruitment period was from February 27 to May 30, 2024, and data collection took place from March 18 to June 7, 2024.

### Participants

Sample size was estimated a priori for a repeated-measures analysis of variance based on a previous SLBT study using MDF [24]. The change in MDF for the ST from start to end served as the reference, with an effect size of d = 0.81. Assuming a correlation of 0.6, the effect size was converted to f = 0.25. With an α = 0.05 and statistical power = 0.80, G*Power indicated 23 participants were required.

To account for poor data quality, twenty-six healthy recreational male athletes were recruited (mean ± standard deviation (SD): age, 21.0 ± 1.9 years; height, 174.1 ± 6.2 cm; mass, 69.4 ± 11.6 kg). Inclusion criteria were age ≥ 18 and regularly resistance training and sprinting (≥ 3 h/week). Exclusion criteria included a history of HSI, and any surgical history of the lower limbs or lumbar region, or current injuries at the time of testing. All participants were informed of the study's benefits and risks and provided written informed consent before participating. This study was conducted with the approval of the institutional research ethics committee (2023–039) and conducted in accordance with the Declaration of Helsinki.

### Procedure

**Fig. 1:**
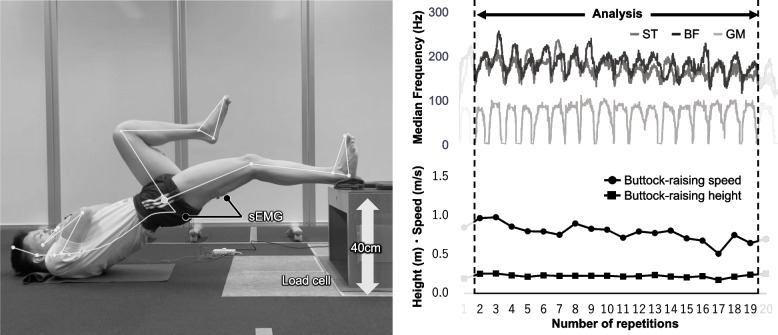
MS-SLBT method and data calculation. MS-SLBT, Maximum-Speed Single-leg bridge test; ST, semitendinosus; BF, biceps femoris; GM, gluteus maximus

Data collection began with a standardized warm-up (3 min of dynamic stretching and 2 min of jogging). Measurements were obtained from the dominant leg (the one used when kicking a ball), starting with 3 SLBT repetitions for familiarization, then 5 practice MS-SLBT repetitions, followed by a 20-repetition MS-SLBT trial as the main measure to assess hamstring muscle endurance (Fig. 1). Participants placed their heel on a 40-cm platform with their knee flexed at 30° which was measured before each trial using a goniometer (GS-100, OG Wellness Inc., Japan). They were instructed to "push down through the heel and raise the buttock as fast and high as possible" [21]. In the lowering phase, participants were instructed to immediately lower their buttock without resistance. To prevent excessive rest during the 20 repetitions trials, a metronome set at 40 bpm was used to guide the timing of each repetition, ensuring one buttock-raising cycle every 1.5 s. Participants were carefully instructed to avoid compensatory motions, including knee extension, lumbar extension, and tibial rotation throughout the task. Movement quality was controlled through verbal instruction only, and visual inspection of the above compensatory movements.

### Measurements

#### Electromyographic measurements

sEMG was used to record muscle activation of the ST, BF, and gluteus maximus (GM) during the MS-SLBT. Before electrode placement, the participants’ skin was shaved, lightly abraded with an abrasive paste, and cleaned with a cotton-alcohol wipe by the examiner. Electrodes (Biometrics Ltd., UK) (37 mm × 20 mm × 6 mm) were placed parallel to the direction of the muscle fibers in positions specified according to the Surface Electromyography for the Noninvasive Assessment of Muscles guidelines and secured with tape to minimize motion artifacts [23, 25]. After warm-up, but prior to the MS-SLBT performance, all participants performed MVIC using a procedure that followed Noraxon's guidelines, with the participant in the prone position on an examination table [26]. The quality of the sEMG signals for each channel was visually inspected on the LabChart 7.3.7 (ADInstruments Inc., Australia) display, and channels were accepted only when baseline noise was low and clear sEMG bursts were observed during light contractions. After confirming the quality, the participants performed two warm-up contractions, followed by three 3–4 s MVIC trials for the hamstrings and GM, with 30 s rest intervals between trials. For the hamstrings, the participants fixed their knees at 30° and secured the distal leg with a band, then performed MVIC in the direction of knee flexion; three trials included neutral, external, and internal tibial rotation, since tibial rotation affects hamstring muscle activation [27]. For GM, the distal thigh was resisted with a band at 90° knee flexion and neutral hip flexion, and MVIC was performed in the direction of hip extension.

#### Motion characteristics

Motion characteristics assessed included the buttock-raising height, buttock-raising speed, and heel-bearing force. During the MS-SLBT, videos were taken from the sagittal plane with an iPhone® that was positioned 220 cm from the participant, with the height from the ground to the lens at 50 cm. The peak buttock-raising height and speed were evaluated using an AI-based marker-less motion-capture analysis app (SPLYZA MOTION®, SPLYZA Inc., Japan). This app computes the coordinates, angles, and speed of each body part using proprietary algorithms and has demonstrated moderate coefficients of variation (11%) for buttock-raising speed based on three consecutive repetitions within a single session [21].

The heel-bearing force was measured using a load cell (Advanced Mechanical Technology Inc., USA) to determine the vertical components of the force, with a value set to zero when the heel was at rest on the platform. For each repetition, the peak heel-bearing force was defined as the highest value recorded during the buttock-raising phase.

### Data analysis

All sEMG and load cell data were sampled at 1000 Hz using a 16-bit PowerLab 8/30 26 T AD unit and analyzed using LabChart 7.3.7. The load cell data were filtered using a 4th-order zero-lag low-pass Butterworth filter with a cutoff frequency of 15 Hz to reduce noise [28]. Muscle fatigue was assessed by evaluating the MDF and amplitude. The MDF was calculated from the raw sEMG data using a custom MATLAB 2024a script (MathWorks Inc, USA). Briefly, this method involves a fast Fourier transform algorithm to compute the power spectrum and divides the data into 128 ms windows. For each window, the cumulative power spectrum is calculated, and the frequency at 50% of cumulative power was defined as the MDF. The amplitude was determined by filtering the sEMG data with a 20–500 Hz bandpass filter and smoothing the signal using the root mean square method with a 50-ms window. The amplitudes were normalized to the maximum amplitude observed during the MVIC trial, defined as 100% MVIC, and expressed as a percentage (%MVIC). All measurements were derived from the maximum value observed during the buttock-raising phase of each MS-SLBT repetition. Based on prior studies and to avoid momentum effects often present in the first and last repetitions [21], we excluded these two trials and evaluated the subsequent 18 repetitions (2nd–19th).

### Statistical analysis

One participant was excluded due to sEMG recording error, resulting in 25 participants being included in the final analysis. Statistical analysis was performed using the JMP Pro 18 software (SAS Institute Inc., USA). Participant characteristics are presented as mean and SD, and the experimental results in the figures are displayed as the mean and standard error of the mean. To identify which of the assessed muscles shows the greatest fatigue during the MS-SLBT, we compared the changes in MDF and amplitude between the three muscles across repetitions using a linear mixed-effects model (LMM) with fixed effects for muscle, repetition, and their interaction, and random intercepts and slopes for each participant. Planned contrasts within the LMM framework were applied for pairwise slope comparisons between muscles, with the significance level adjusted to *p* < 0.0167 using Bonferroni correction for the three muscle comparisons. Changes in MDF and amplitude within each muscle, as well as changes in buttock-raising height, buttock-raising speed, and heel-bearing force, from 2–19 repetitions were analyzed using LMM with repetition as a fixed effect and participant as a random effect. When significant differences were detected, Dunnett's test was used for post hoc comparisons, with the second repetition set as the control. In addition, Cohen’s d was calculated to quantify the magnitude of change between the 2nd and 19th repetitions. Effect sizes were interpreted according to Hopkins et al. (2009) as follows: 0.2 (small), 0.6 (moderate), 1.2 (large), 2.0 (very large), and 4.0 (extremely large) [29]. The significance level for all analyses was set at *p* < 0.05, except for those using a corrected *p*-value.

## Results

**Fig. 2:**
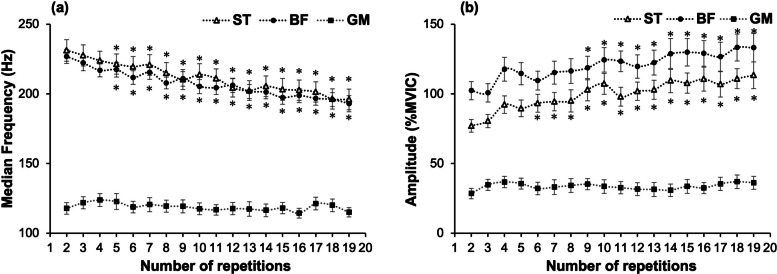
Changes in muscle activity during 20 repetitions of the MS-SLBT. **a** Median frequency; **b** Amplitude. Data points represent the mean values; error bars indicate the standard error. The asterisk (*) indicates a significant difference (*p* < 0.05) compared with the second repetition, as determined by Dunnett's test. MS-SLBT, maximum-speed single-leg bridge test; ST, semitendinosus; BF, biceps femoris; GM, gluteus maximus

The interaction between muscle and repetition for MDF was significant (*p* < 0.01, F = 27.01) (Fig. 2). Within each muscle, a significant decrease over time was found in the ST and BF (*p* < 0.01), but not in the GM (*p* = 0.57). The decrease from the 2nd to the 19th repetition showed a moderate effect size in the ST (d = 0.93) and a large effect size in the BF (d = 1.26). Dunnett's test showed that, compared with the 2nd repetition, MDF significantly decreased from the 5th repetition onward in both ST and BF (*p* < 0.05). The ST and BF showed a significantly greater decrease in MDF than the GM (*p* < 0.01), whereas no significant difference was observed between the ST and BF (*p* = 0.44). The interaction between muscle and repetition for amplitude was significant (*p* < 0.01, F = 13.60). Within each muscle, a significant increase over time was found in the ST and BF (*p* < 0.01), but not in the GM (*p* = 0.79). From the 2nd to the 19th repetition, amplitude increased with moderate effect sizes in the ST (d = 0.97) and BF (d = 0.72). Dunnett's test showed that amplitude increased from the 6th and 9th repetitions onward in ST and BF, respectively (*p* < 0.05). In slope comparisons between muscles, significant differences were observed between GM and ST, and between GM and BF (*p* < 0.01), whereas no significant difference was found between ST and BF (*p* = 0.65).

**Fig. 3:**
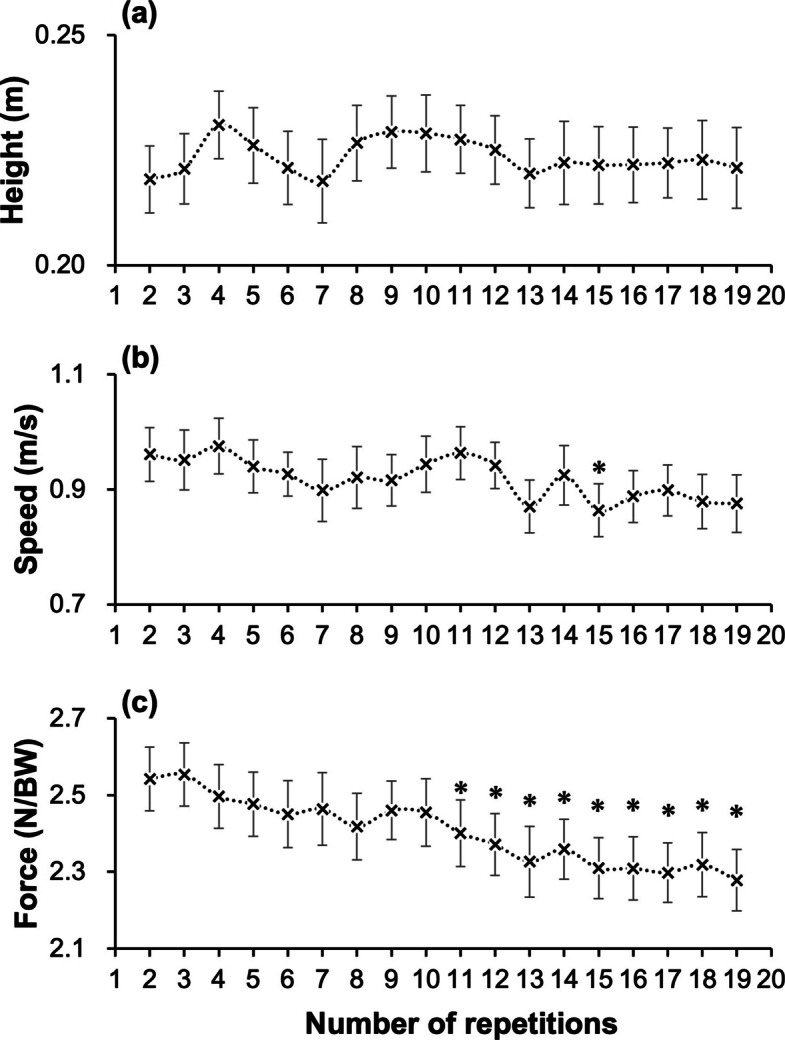
Changes in motion characteristics during 20 repetitions of the MS-SLBT. **a** Buttock-raising height; **b** Buttock-raising speed; **c** Heel-bearing force. Data points represent the mean values; error bars indicate the standard error. The asterisk (*) indicates a significant difference (*p* < 0.05) compared with the 2nd repetition, as determined by Dunnett's test. MS-SLBT, maximum-speed single-leg bridge test

Motion characteristics evaluated using the peak of each repetition showed no significant differences in the buttock-raising height from 2–19 repetitions (*p* = 0.73, F = 0.12). In contrast, the buttock-raising speed (*p* < 0.01, F = 8.09) and heel-bearing force (*p* < 0.01, F = 54.29) decreased significantly (Fig. 3). From the 2nd to the 19th repetition, the decrease in buttock-raising speed showed a small effect size (d = 0.35), whereas heel-bearing force showed a moderate effect size (d = 0.65). Post hoc multiple comparisons revealed that the buttock-raising speed significantly decreased at the 15th repetition compared to that at the 2nd repetition (*p* < 0.05). The heel-bearing force significantly decreased from the 11th repetition onward as compared to the 2nd repetition (*p* < 0.05).

## Discussion

The results of this study demonstrated that 20 repetitions of the MS-SLBT significantly decreased the MDF of the ST and BF by 15.2% and 14.9%, respectively, from start to end (2nd to 19th repetition), whereas no change was observed in the GM. Furthermore, there were also significant reductions in buttock-raising speed and heel-bearing force by 8.9% and 10.4%, respectively, without changes in the buttock-raising height.

### Muscle activation

In this study, the MDF of the ST and BF significantly decreased, whereas the amplitude increased, which generally reflects the progression of muscle fatigue [22, 30]. Muscle fatigue leads to a reduction in muscle fiber conduction velocity, primarily due to metabolite accumulation and ion imbalance during repeated contractions [31]. This reduction shifts the sEMG signal’s power spectrum toward lower frequencies, thereby lowering the MDF [31]. An increase in the amplitude reflects a compensatory recruitment of additional motor units to counteract the decline in force output caused by muscle fatigue [30].

In a conventional SLBT repeated until exhaustion, the MDF decreases by 18.0% for the ST and by 17.7% for the BF, while amplitude increases by 11.5% for the ST and by 16.7% for the BF [24]. Similarly, in the present MS-SLBT, the MDF decreased by 15.2% for the ST and by 14.9% for the BF, whereas amplitude increased by 47.1% for the ST and by 30.1% for the BF. These comparable MDF reductions suggest that the MS-SLBT can induce a level of hamstring muscle fatigue similar to the conventional SLBT, despite requiring only 20 repetitions. Multiple comparisons further revealed that fatigue developed early, with significant MDF decreases for the ST and BF occurring already after the 5th repetition, followed by compensatory motor unit recruitment reflected in amplitude increases after the 6th and 9th repetitions, respectively. Interestingly, ST and BF amplitude increased more in the MS-SLBT as compared to the conventional SLBT. This may speculatively reflect the greater fatigue induced by the high-speed contractions, necessitating the additional recruitment of large motor units associated with type II fibers, as opposed to additional recruitment of type I fibers in the SLBT due to the lower contraction speed. Muscles with a higher proportion of Type II fibers exhibit higher initial MDF values and more substantial decreases during contraction [32, 33], reflecting the greater fatigue induced by high-speed contractions that recruit large motor units associated with Type II fibers. This aligns with our findings showing that the relative MDF decrease was substantially greater for the ST and BF than for the GM [34], which is consistent with previous reports indicating that muscles with a higher proportion of Type II fibers show larger reductions during repeated contractions. However, due to methodological differences, direct comparison with previous studies and interpretations related to muscle fiber type should be made with caution. These results suggest that the MS-SLBT more effectively induces high-frequency fatigue in the ST and BF compared to the GM, thereby selectively and efficiently eliciting hamstring muscle fatigue within only 20 repetitions.

### Motion characteristics

The muscle fatigue—as inferred from sEMG measures—observed in the ST and BF likely contributed to kinematic and kinetic changes, such as reductions in buttock-raising speed and heel-bearing force, rather than changes in buttock-raising height. Indeed, heel-bearing force during the MS-SLBT decreased by approximately 18.4 N from the 2nd to the 19th repetition. Recent evidence demonstrated that isometric heel-bearing force measured in a similar position could predict the risk of HSI, with injured group showed an average of 26 N lower force and each 10 N increase in force was associated with a 7.4% reduction in the risk of HSI [14]. This suggests that this decline may also be relevant to the risk of HSI. This finding likely reflects reduced mechanical output of the hamstring muscles, which act as hip extensors and knee flexors. Given that the body mass remained constant during the test, a reduction in heel-bearing force necessarily indicates a proportional reduction in acceleration according to Newton’s second law (F = m × a), suggesting a diminished capacity to generate rapid hip extension. As participants were instructed to push down through the heel while maintaining knee flexion and while extending the hip as fast as possible, the reduced force likely reflects a decreased ability to sustain hip extension velocity in a knee flexed position. Taken together, these findings suggest that muscle fatigue observed in the ST and BF during the early repetitions may have contributed to a reduced ability to generate fast hip extension in a knee-flexed position in later repetitions, leading to an inability to maintain acceleration in hip extension and, consequently, a decrease in buttock-raising speed.

However, no change in the buttock-raising height was observed. While the conventional SLBT uses height to assess fatigue, our finding suggests speed may be a more sensitive indicator than height for detecting fatigue. This may be because muscles with fewer fast-twitch fibers, such as the GM and adductor magnus [35], help maintain height during hip-extension movements, but their slower fiber type may not allow maintenance of movement speed. Such a finding is in line with previous studies showing measures of explosive force generally better capture fatigue than measures of maximal force [36, 37]. Although heel-bearing force may be the most sensitive indicator of fatigue detection given its more consistent decrease (Fig. 3), it requires specialized equipment such as a load cell or handheld dynamometer [38]; therefore, using buttock-raising speed as a surrogate could enhance the practicality and applicability of this assessment.

Furthermore, a case report using the MS-SLBT demonstrated that hamstring functional deficits could be detected through lower buttock-raising height on the injured side [39]. Interestingly, this difference was evident within the first few repetitions rather than across the full set, suggesting that the MS-SLBT may also be capable of detecting baseline functional deficits, not only endurance-related decline. To properly interpret endurance measures, sufficient initial output during early repetitions is required. Therefore, when testing individuals with a history of HSI or current injury, changes in height may be observed, or alterations in speed and heel-bearing force may occur earlier, resulting in findings that differ from those in the present study. Future studies should therefore establish normative reference values for buttock-raising height and speed, as well as endurance-related decline rates, and determine whether these parameters can predict HSI occurrence or guide safe return-to-play decisions.

### Clinical implications

By allowing hamstring muscle endurance to be evaluated within 30 s with 20 repetitions, the MS-SLBT offers significant benefits for both test subjects and testers. Conventional muscle endurance tests often require longer durations, taking approximately 4 min 30 s to complete [5], which makes them less practical in sports settings or clinical environments. Furthermore, maintaining athlete motivation during prolonged testing can be challenging as performance during exhaustive protocols may be influenced by psychological factors rather than purely physiological fatigue [40, 41]. The MS-SLBT requires only a 40 cm platform, which can be substituted with common clinical equipment, such as chairs or training benches, making this test highly versatile and easy to implement in various settings. Furthermore, a case report using the MS-SLBT as the sole intervention exercise revealed significant improvements in buttock-raising performance after 1–2 months [39], indicating the potential of this simple assessment method as an exercise tool.

### Limitations

This study had some limitations. The participants were healthy university students who were part of recreational athletic teams, which makes it unclear whether the findings are applicable to female participants [42] or elite athletes. Regarding sex differences, although women generally exhibit greater fatigue resistance, this advantage tends to diminish during high-intensity exercise [43], suggesting that the present findings may still be applicable to female participants. In elite athletes, neuromuscular fatigue responses are similar to those of recreationally active individuals, although the magnitude may differ [44]. Additionally, sEMG is susceptible to crosstalk between adjacent muscles [45], and during dynamic tasks, slight electrode displacement due to skin movement may affect the recorded signals [46]. The markerless motion analysis system (SPLYZA Motion®) used in this study has not yet been fully validated or reliability-tested in peer-reviewed literature. Because this system uses a two-dimensional analysis, we carefully verified the consistency between the visual motion angles on the video and the automatically calculated data. Furthermore, only the vertical component of the ground reaction force was analyzed in this study, which is in line with previous studies using isometric tests in the same position that also analyzed only the vertical force [14, 38], ensuring methodological consistency with established protocols. Nevertheless, the assessment of anterior–posterior and mediolateral components may provide additional insights into compensatory movements. Finally, the findings of this study represent normative values for healthy male individuals, and the relationship between these values and HSI remains unclear. Future research should compare individuals with and without HSI and use prospective cohort studies to examine whether MS-SLBT performance is associated with future HSI.

## Conclusion

Twenty repetitions of the MS-SLBT, which can be completed in just 30 s, demonstrated reductions in MDF and increases in amplitude for the ST and BF, as well as decreased buttock-raising speed and heel-bearing force during the later repetitions. These findings highlight the potential of the MS-SLBT as a specific and practical tool for evaluating hamstring muscle endurance.

## Data Availability

The data supporting the findings of this study are not publicly available due to ethical restrictions but may be shared by the corresponding author upon justified request.

## Abbreviations

BF: Biceps femoris
GM: Gluteus maximus
HSI: Hamstring strain injuries
LMM: Linear mixed-effects model
MDF: Median frequency
MS-SLBT: Maximum-speed single-leg bridge test
MVIC: Maximal voluntary isometric contraction
sEMG: Surface electromyography
SLBT: Single-leg bridge test
ST: Semitendinosus
